# Vaccine Side Effects Following COVID-19 Vaccination Among the Residents of the UAE—An Observational Study

**DOI:** 10.3389/fpubh.2022.876336

**Published:** 2022-05-06

**Authors:** Subhashini Ganesan, Latifa Mohammad Baynouna Al Ketbi, Nawal Al Kaabi, Mohammed Al Mansoori, Noura Nasser Al Maskari, Mariam Saif Al Shamsi, Aysha Saeed Alderei, Hamada Nasser El Eissaee, Rudina Mubarak Al Ketbi, Noura Saeed Al Shamsi, Khuloud Mohammed Saleh, Aysha Fahad Al Blooshi, Flavia Martinez Cantarutti, Katherine Warren, Faheem Ahamed, Walid Zaher

**Affiliations:** ^1^G42 Healthcare, Abu Dhabi, United Arab Emirates; ^2^Insights Research Organization and Solutions, Abu Dhabi, United Arab Emirates; ^3^Sheikh Khalifa Medical City, SEHA, Abu Dhabi, United Arab Emirates; ^4^College of Medicine and Health Sciences, Khalifa University, Abu Dhabi, United Arab Emirates; ^5^Ambulatory Healthcare Services, SEHA, Abu Dhabi, United Arab Emirates; ^6^College of Medicine and Health Sciences, UAE University, Abu Dhabi, United Arab Emirates

**Keywords:** SARS-CoV-2, COVID-19, adverse (side) effects, Pfizer-BioNTech vaccine, Sinopharm vaccine

## Abstract

COVID-19 vaccines have proven to be very safe in the clinical trials, however, there is less evidence comparing the safety of these vaccines in real-world settings. Therefore, we aim to investigate the nature and severity of the adverse effects reported and the differences based on the type of vaccine received. A survey was conducted among 1,878 adult (≥18 years) COVID-19 vaccine recipients through online survey platforms and telephonic interviews during March to September 2021. The factors potentially associated with the reported side effects like age, gender, ethnicity, comorbidities, and previous COVID-19 infection were analyzed based on the type of vaccine received. Differences in adverse events and the severity were compared between inactivated and mRNA vaccine recipients. The major adverse effects reported by the COVID-19 vaccine recipients were pain at the site of injection, fatigue and drowsiness, and headache followed by joint/muscle pain. The adverse effects were more common among recipients of mRNA Pfizer-BioNTech vaccine than among recipients of inactive Sinopharm vaccine with the odds ratio of 1.39 (95% CI 1.14–1.68). The average number of adverse effects reported between individuals who had received Sinopharm and Pfizer-BioNTech vaccines was 1.61 ± 2.08 and 2.20 ± 2.58, respectively, and the difference was statistically significant (*p* <0.001). Severe adverse effects after COVID-19 vaccinations were rare and 95% of the adverse effects reported after either an inactivated or mRNA vaccine were mild requiring no or home-based treatment. The study found that individuals less than 55 years of age, female gender, with history of one or more comorbid conditions, who had received mRNA Pfizer- BioNTech vaccine, and with history of COVID-19 infections are at higher odds of developing an adverse effect post COVID-19 vaccination compared to the others.

## Introduction

In the world's fight against the COVID-19 pandemic, vaccines emerged as the greatest savior for humankind and many vaccine candidates were developed and entered clinical trials in early 2020. Many of these vaccines obtained emergency approval and governments around the world started vaccination campaigns against COVID-19 (1, 2).

The United Arab Emirates (UAE) government had successfully completed the vaccination trial for the BBIBP-CorV or Sinopharm vaccine which was approved for general public in December 2020 (3). Since then, vigorous vaccination of the population was undertaken and the UAE tops the world in the vaccine distribution rate per 100 people (4)and has effectively vaccinated more than 90% of the population so far (5). Apart from Sinopharm, the UAE government has also approved mRNA Pfizer-BioNTech, adenovirus-based AstraZeneca, and Sputnik V and other vaccines and made them accessible for all people in the UAE.

The worldwide vaccination coverage for COVID-19 vaccination is still low and as of 24 November 2021, only 42% of the world population have received initial two doses of COVID-19 vaccination (6). Data published from the vaccine trials have demonstrated that the adverse effects of the COVID-19 vaccine most commonly include fever, fatigue, muscle pain, joint pain, and headache and serious adverse events were rarely reported (7–10). In the Sinopharm inactivated vaccine trial, the most common adverse reaction observed was injection site pain followed by fever and all the adverse reactions reported were mild and self-limiting and did not require any treatment (7). The Pfizer-BioNTech mRNA vaccine trial has reported that the most common adverse effect was mild to moderate fatigue and headache (8). Similarly, AstraZeneca and Sputnik vaccine trials have also reported only mild/moderate side effects (9, 10).

Global safety studies on vaccine reactogenicity have shown that the mRNA COVID-19 vaccine recipients have reported injection site or local reactions more frequently than systemic reactions and serious adverse events were rare (11, 12). Younger population and females have reported more side effects than the others (13). Despite published safety data on COVID-19 vaccines, people around the globe have expressed their concerns and hesitancy in vaccination, and studies have shown that the intention to get vaccinated is associated with positive vaccination beliefs (14). Surveys have shown that people worry about the potential adverse effects of the COVID-19 vaccine which they believe would be worse than the disease itself (15, 16). Even among healthcare professionals, vaccine acceptance has been found to be sub-optimal with doubts about vaccine safety, the quality control, and potential adverse effects given its rapid development (17, 18).

A survey done in the UAE on vaccine perception for COVID-19 vaccine showed that safety of the vaccine with no major side effects emerged as the greatest motivating factor to get vaccinated (19). As vaccine-related fears on safety and side effects play a role in determining the decision regarding vaccination, studies are conducted to monitor the safety of COVID-19 vaccines globally (20). All these reports establish that the major concern for people to get vaccinated is the side effects or adverse events following COVID-19 vaccination.

Therefore, this survey would give an insight into the actual adverse effects experienced by the recipients of the COVID-19 vaccine and will help us understand the nature of the adverse effects of these different vaccines. This insight would also help instill confidence in people on getting vaccinated against COVID-19.

## Methods

### Study Design and Study Setting

A cross-sectional study based on an online survey and telephonic interviews was conducted between 14 March 2021 and 4 September 2021 among the residents of the UAE. The survey was designed to identify the side effects reported after receiving a COVID-19 vaccination and no personal identification details were collected. An electronic consent was obtained during the online survey and only participants who agreed entered the survey. Participants in telephonic interviews also consented orally before they were presented with the survey. The study was approved by the Medical Research Department, DOH, Abu Dhabi, UAE (approval number: DOH/CVDC/2021/329).

### Study Participants

Participants from all nationalities, gender, and age 18 years and above, who had received at least one dose of the COVID-19 vaccination, who were able to give consent and were currently residing in the UAE were included in the survey. The survey form was designed using Google Forms and the participants were approached through social media platforms. A total of 744 participants consented and completed the survey online. Apart from this, telephonic interviews were conducted and the participants were selected randomly from the vaccine database of those who had received the COVID-19 vaccination during the period of the study available at the Ambulatory Healthcare Services, SEHA. This is Abu Dhabi's largest health services provider, which operates all public hospitals and clinics in the emirate of Abu Dhabi. It provides vaccination against COVID-19, manages vaccine-related complications, and operates all COVID-19 dedicated hospitals in Abu Dhabi. Stratified random sampling was used to ensure good representation and samples were stratified based on age group and type of vaccine received. The selected participants were contacted, and among those who were willing to participate, the survey was completed through a telephone interview. A total of 1,796 vaccine recipients were selected from the database based on the stratification criteria and were approached. The response rate was 63.1% with 1,134 completing the interview. Of the others who did not participate, 4.7% refused to participate, 20% did not answer the phone calls, and the rest were identified as having wrong numbers or having language barriers. The interviews were done by family medicine residents who were trained for this purpose. Therefore, a total of 1,878 participants completed the survey, of which 1,134 participants were interviewed through a telephonic survey and 744 participants completed the online survey form.

### The Survey Tool

The questionnaire was designed to capture the reactogenicity to the COVID-19 vaccine and the factors that were related to the development of the vaccine's side effects. Based on the literature review of the side effects reported post-COVID-19 vaccination (8–14), the adverse effects were listed in our survey and avenues to add additional side effects were provided. The factors that were studied in the literature and found to be associated with the development of the side effects were captured by designing specific questions on age, gender, nationality, educational status, monthly income, comorbid conditions, previous history of COVID-19 infection, type of COVID-19 vaccine received, and the number of doses received to elicit the responses (11–13, 21).

The questionnaire was prepared both in English and Arabic languages. The Arabic translation was done by language experts and back-translated by two native speakers to understand any discrepancies and then corrected and approved by the language experts.

The construct validity of the questionnaire was established by four experts with epidemiology, public health, and family medicine background and experience in vaccine trials and vaccination programs. The questionnaire was pilot tested among 5 participants to understand the feasibility and was refined based on feedback.

The survey had a screening question on vaccination which screened all participants who were not vaccinated. Only the participants who had received at least one dose of the COVID-19 vaccine were presented further for the survey.

The first part of the survey included questions on demographic variables like age, sex, education, and nationality. The second part of the survey included questions on comorbid conditions like diabetes, hypertension, chronic lung diseases, cardiovascular, cancer, autoimmune diseases, and other chronic diseases. This section also included a question on the previous history of allergies and associated immunodeficiency or taking medication like high-dose corticosteroids, immunosuppressants, or cancer medicines to rule out any immunocompromised state. The third section included questions on the previous infection with COVID-19 and the severity of the infection. The fourth section of the survey was on vaccination, the type of vaccination taken against COVID-19, the number of doses taken, and the adverse effects experienced after vaccination for COVID-19. The final section of the survey was only for people who had experienced any adverse effects. This section included more questions to understand the nature of adverse effects. Questions on doses of vaccination after which they developed adverse effects and the severity of the adverse effects were included. Participants were asked to grade the severity of side effects by using a Likerts scale of 1–10, where 1 denotes “mild symptoms” and 10 denotes “extremely severe symptoms”. To assess severity more objectively, we also asked the participants whether the side effects required any treatment, if so, was it home-based treatment, consultation with a health professional, or hospital-based treatment.

The age of the participants was stratified into three groups <35 years, 35–54 years, and 55 years and above, which was based on previous literature and the age stratification widely employed in the studies reviewed on systematic review and metanalysis of the impact of age difference on COVID-19 vaccine safety (12, 22). The adverse effects were classified into local and systemic side effects for analysis. The local side effects included injection site reactions like pain at the site of vaccination, redness, swelling/lymph node enlargement, and itch. The systemic side effects included fever, headache, muscle, and joint pain, flu-like symptoms, etc.

### Sample Size

The study was conducted only among residents of the UAE, which has a population of approximately 10 million people, and at the time of the study, 4 million doses were administered, that is approximately 40% of the total population had received at least one dose of their vaccination against COVID-19 (23). Hence, with 40 % vaccination coverage with power 80% and relative precision of 10%, the sample size was calculated to be 1,200 which accommodated a non-response rate of 50%.

### Statistical Analysis

The adverse effects experienced post-COVID-19 vaccination are expressed in percentage (%) and a chi-square test was used to test the significance of the difference between the demographics and other variables of interest (comorbid conditions, previous history of COVID-19 infection, etc.) against adverse effects. The difference in adverse effects based on the type of vaccine and doses was also compared using the chi-square test and the odds ratio was calculated. *p* value < 0.05 was considered statistically significant. All statistical analyses were done using SPSS software version 28.0.

## Results

Of the 1,878 participants who completed our survey, 940 (50 %) belonged to the 35–55-year age group, 1,151 (61.3%) were females, 526 (28%) had associated comorbid conditions, and 332(17.7%) had a history of a previous infection with COVID-19. The demographical details of all the participants are described in Supplementary Table S1.

A total of 941 (50.1%) people received inactivated Sinopharm vaccine, 890 (47.4%) received mRNA Pfizer-BioNTech vaccine, and 11 (0.6%) received Adenovirus vector AstraZeneca vaccine; 36 (1.9%) were not aware of the details of the vaccine received. Among the study participants, 1,795 (95.6%) had received two or more doses of COVID-19 vaccination and 83 (4.4%) had received one dose of vaccination.

### Adverse Reactions Following COVID-19 Vaccination

**Figure 1:**
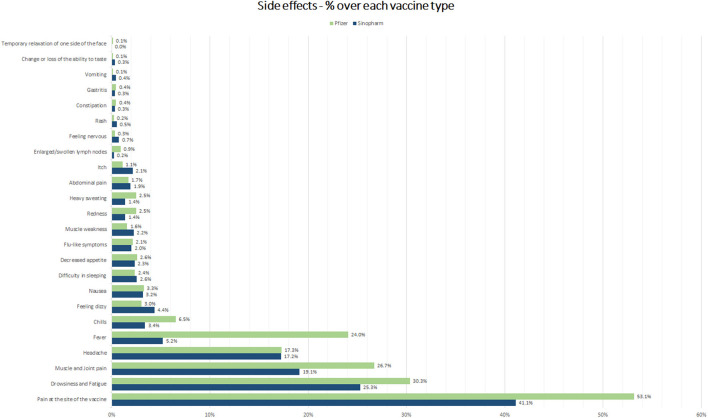
Adverse effects reported post-COVID-19 vaccination.

A total of 1,217 (64.8%) study participants reported one or more side effects following COVID-19 vaccination. The major adverse effects reported by the COVID-19 vaccine recipients were pain at the site of injection (47%), fatigue and drowsiness (28.2%), and joint/muscle pain (23.1%) followed by headache (17.7%) and fever (14.4%) (Supplementary Table S2). The percentage of side effects reported based on the type of vaccine is shown in Figure 1. Since most of the study participants either received an inactivated Sinopharm or mRNA Pfizer-BioNTech vaccine and only a negligible percentage of the people had received other vaccines further analyses were done on these two vaccine groups only.

### Adverse Effects Following Inactivated Sinopharm Vaccine and the Factors Associated

A total of 574 (61%) of the Sinopharm vaccine recipients reported adverse effects following vaccination. The analysis of various factors related to reactogenicity among the participants who received the Sinopharm vaccine found that younger age group (<55 years), female gender, individuals with higher educational status (graduate/postgraduate), and people with associated comorbidities reported a statistically significant higher percentage of adverse effects than the others (Table 1).

**Table 1:** Association of factors with the adverse effects after receiving the inactive Sinopharm vaccine.

**Factors**		**Total (*n*)**	**Adverse effects**	**Odds ratio**	***P*-value**
Age group	<35 years	366	229 (62.6%)	1.98 (1.18–3.32)	<0.001
	35–54 years	505	313 (62.0%)	1.93 (1.17–3.20)	
	≥55 years	70	32 (45.7%)	1	
Gender	Male	321	161 (50.2%)	1.98 (1.50–2.61)	<0.001
	Female	620	413 (66.6%)		
Educational status	Highschool/diploma	273	136 (49.8%)	1.45 (1.24–1.70)	<0.001
	Graduate/post graduate	668	438 (65.6%)		
Income	≤ 10,000 AED	275	169 (61.5%)	1.129 (0.93–1.36)	0.239
	Above 10,000 AED	495	326 (65.9%)		
Ethnicity	Emirati	529	314 (59.4%)	0.854 (0.65–1.11)	0.253
	Non-Emirati	412	260 (63.1%)		
Comorbidities	With comorbidities	424	289 (68.2%)	1.74 (1.33–2.27)	<0.001
	No comorbidities	517	285 (55.1%)		
Previous COVID-19 infection	Yes	154	98 (63.6%)	1.14 (0.79–1.63)	0.472
	No	787	476 (60.5%)		

### Adverse Effects Following Pfizer-BioNTech (MRNA) Vaccine and the Factors Associated

A total of 610 (68.5%) reported adverse effects following the Pfizer-BioNTech vaccination. The association of demographic variables, comorbid conditions, and previous history of COVID-19 infection with the adverse effects following the Pfizer-BioNTech vaccine showed that age of <35 years, higher educational status, higher income, individuals with comorbidities, and individuals who had a history of previous infection with COVID-19 reported a statistically significant higher percentage of adverse effects than the others (Table 2).

**Table 2:** Association of the risk factors with adverse effects after receiving the mRNA Pfizer vaccine.

**Factors**		**Total (*n*)**	**Side effects**	**Odds ratio**	***P*-value**
Age group	<35 years	210	118 (56.2%)	0.539 (0.369–0.787)	0.001
	35–54 years	413	304 (73.6%)	1.172 (0.832–1.649)	0.363
	≥55 years	267	188 (70.4 %)	1	
Gender	Male	391	264 (67.5%)	1.088 (0.818–1.446)	0.562
	Female	499	346 (69.3%)		
Educational status	Highschool/diploma	431	278 (64.5%)	1.283 (1.056–1.559)	0.014
	Graduate/post graduate	459	332 (72.3%)		
Income status	≤ 10,000 AED	278	174 (62.6%)	1.568 (1.204–2.041)	<0.001
	Above 10,000 AED	264	201 (76.1%)		
Ethnicity	Emirati	352	252 (71.6%)	1.267 (0.945–1.698)	0.121
	Non-Emirati	538	358 (66.5%)		
Comorbidities	With comorbidities	280	206 (73.6%)	1.419 (1.037–1.943)	0.030
	No comorbidities	610	404 (66.2%)		
Previous COVID-19 infection	Yes	171	129 (75.4%)	1.520 (1.038–2.225)	0.035
	No	719	481 (66.9%)		

### Comparison of Adverse Effects Between Sinopharm and Pfizer-BioNTech Vaccine Recipients

The adverse effects were more common among recipients of the mRNA Pfizer-BioNTech vaccine than among recipients of the inactive Sinopharm vaccine with the odds ratio of 1.39 (95% CI 1.14–1.68); 4 out of 10 participants reported no side effects after receiving the Sinopharm vaccine compared to the Pfizer-BioNTech vaccine recipients among which 3 out of 10 reported no side effects (Supplementary Table S3).

### Vaccine Dose and Adverse Effects

Among the recipients of inactivated Sinopharm vaccine 214 (22.7%), 208 (22.1%) and 128 (13.6%) reported adverse effects after the first, second, and both doses, respectively. Similarly, among the recipients of mRNA Pfizer-BioNTech vaccine, 103 (11.6%), 268 (30.1%), and 232 (26.1%) reported adverse effects after the first, second, and both doses, respectively (Supplementary Table S3).

The first-dose Sinopharm vaccine recipients are 2.9 times more likely to develop adverse effects compared to the first-dose recipients of the Pfizer-BioNTech vaccine (*p* < 0.001), however, after the second dose, recipients of the Pfizer-BioNTech vaccine were 1.4 times more likely to develop an adverse effect than the second-dose Sinopharm vaccine recipients (*p*: 0.007) (Supplementary Table S3).

### Local and Systemic Side Effects

The Sinopharm vaccine recipients reported 393 (41.8%) and 417(44.3%) local and systemic symptoms, respectively. Among the Pfizer vaccine recipients, 478(53.7%) reported local symptoms and 432 (48.5%) reported systemic symptoms after vaccination.

While no statistically significant difference was observed with the local adverse effects reported after the first and second dose of the Sinopharm and Pfizer vaccine recipients, respectively, a statistically significant higher percentage of systemic adverse effects was reported after the first dose in the Sinopharm vaccine recipients (78.5%) compared to the first-dose Pfizer vaccine recipients (64.1%) (odds ratio 2.04 *p*-value-0.007). However, after the second dose of the Pfizer vaccine, recipients reported more systemic side effects (86.6%), which was statistically significant compared to the systemic side effects reported after the second dose of the Sinopharm vaccine (72.1%) (odds ratio 1.12, *p*-value <0.001) (Supplementary Table S3).

### Age and Vaccine Adverse Effects

Based on age group, among people <35 years of age, there was no significant difference in the occurrence of the side effects based on the type of vaccine but among people in the 35–54 age group and in the age group ≥55 years, those who received the Pfizer-BioNTech vaccine were 1.44 (95% CI 1.18,1.75) and 1.83 (95% CI 1.38, 2.43) times more at risk of having an adverse effect than those who received the Sinopharm vaccine (Supplementary Table S4). Similar results were seen when the average number of side effects reported were considered among the different age groups based on the vaccine received.

### Number of Sides Effects Reported

Considering the number of side effects reported, 54.5 and 6.5% of the inactive vaccine recipients reported 1–5 side effects and 6–14 side effects, respectively, compared to the mRNA vaccine recipients of which 55.9 and 12.7% reported 1–5 side effects and 6–14 side effects, respectively. The average number of adverse effects reported between individuals who had received the Sinopharm and Pfizer-BioNTech vaccine was 1.61 ± 2.08 and 2.20 ± 2.58, respectively, and the difference was statistically significant (*p* < 0.001).

**Figure 2:**
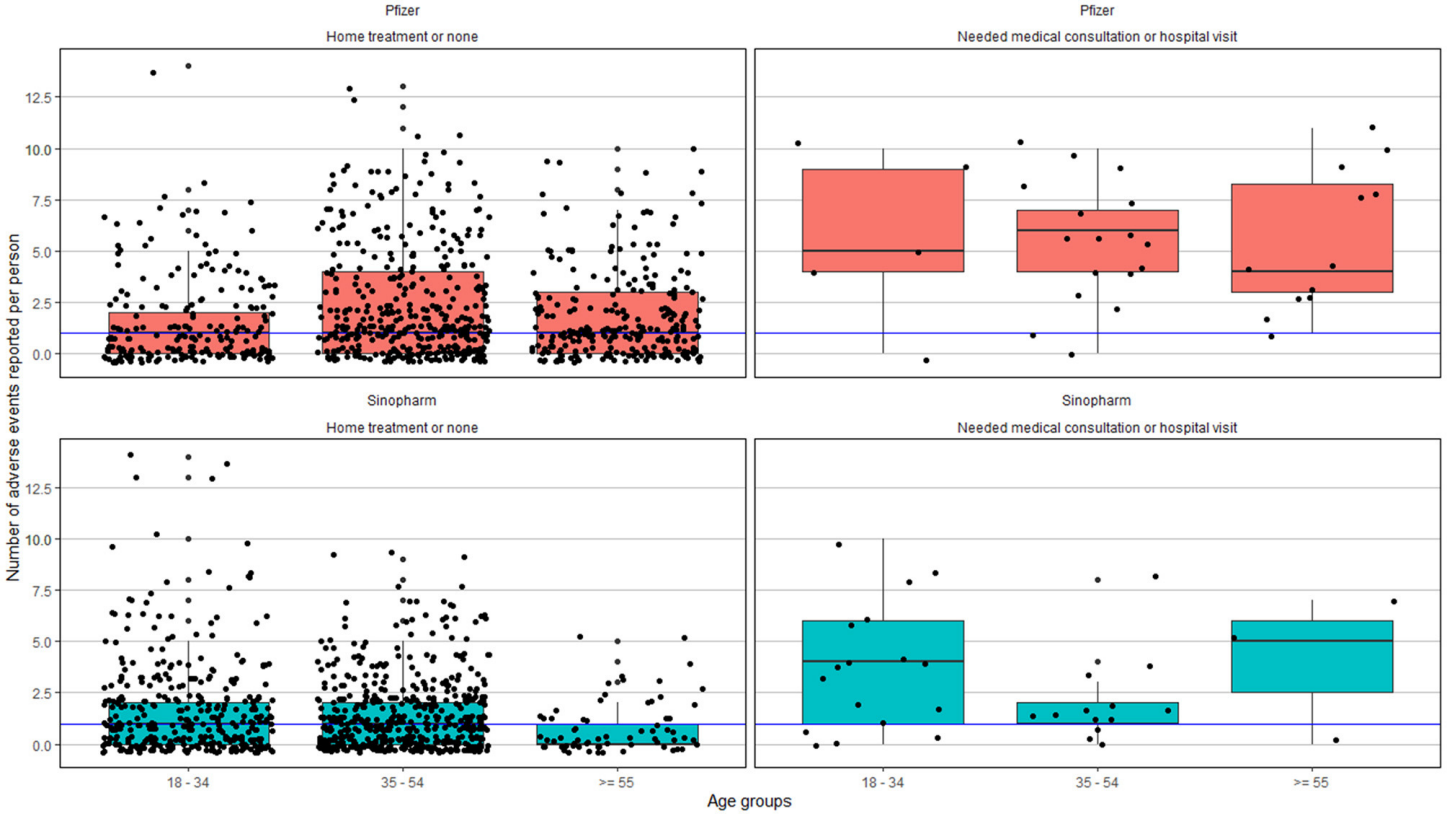
Adverse effects reported based on the age group and severity. The blue line represents the median of the number of adverse events reported across all age groups.

Statistically significant difference was observed among people in the 35–54-years age group and ≥ 55-years age group. Based on the number of side effects reported in the 35–54-years age group and 55-years age group, those who received the Pfizer-BioNTech vaccine on an average reported 3.49± 2.70 and 2.93 ± 2.34 adverse effects, respectively, compared to 2.42 ± 1.76 and 2.18 ±1.55, among those who received the Sinopharm vaccine. No statistically significant difference was observed in the <35 years of age cohort (Figure 2).

### Severity of Side Effects

A total of 1,137 (62.2%) graded the severity of the symptoms, of which 924 (81%) graded it with a score below 5; 1,154 (63.0%) answered the question on the treatment for side effects, of which only 59 (5.0%) required consultation or hospital visit, 1,095 (95.0%) did not require any treatment or only home-based treatments. No severe adverse events were reported (Figure 2 and Supplementary Figure S1).

The observed proportion of adverse effects that required either a doctor's advice or a hospital-based treatment was slightly higher in the age group ≥55 years but no statistical significance was observed between the two vaccine recipients among the different age groups (Supplementary Table S5).

## Discussion

Vaccine reactogenicity represents various local and systemic manifestations because of the inflammatory response to vaccination. The reactogenicity depends on various factors like the host characteristics (age, gender, etc.), type of vaccine, composition, route of administration, and many others (24). Therefore, it is likely that most individuals would exhibit vaccine reaction post-COVID-19 vaccination. The survey shows that around 65% of the study participants experienced some adverse reaction due to the COVID-19 vaccination. None of the study participants reported severe allergic reactions to the COVID-19 vaccines. The most common adverse effects experienced among both the inactivated and the mRNA vaccine recipients were pain at the site of vaccination followed by fever, fatigue, and headache among the mRNA Pfizer-BioNTech recipients and fatigue and headache among the inactivated Sinopharm vaccine recipients, respectively. The rarest adverse effect reported among the Sinopharm vaccine recipients was enlarged swollen lymph nodes and change or loss of taste; while among the mRNA Pfizer-BioNTech vaccine recipients, temporary one-sided facial weakness was reported by one participant followed by enlarged swollen lymph nodes. Similar rare adverse effects were also reported in a study from Saudi Arabia on the Pfizer-BioNTech vaccine recipients (25). The nature of the adverse effects reported were similar to the adverse events mentioned in the safety and efficacy study of the Sinopharm and Pfizer-BioNTech vaccines but the percentage observed in this study was higher than reported in the trials (7, 8). However, other reports on real-world data have reported a similar percentage of adverse effects observed among both inactivated and mRNA vaccine recipients (26–28).

Among the participants who received the Sinopharm vaccine, younger age group (<55 years), female gender, individuals with higher educational status, higher income, and people with comorbidities had reported a statistically significant higher percentage of adverse effects which was again supported by evidence from other studies on the inactivated vaccine (21, 29). Among the Pfizer-BioNTech vaccine recipients, statistically significant associations with the vaccine's adverse effects were observed among participants in the age group of 35 years and above with higher educational status, higher income, individuals with comorbidities, and among individuals who had a history of a previous infection with COVID-19. Studies have demonstrated similar findings where higher vaccine reactogenicity was observed among individuals previously infected with the COVID-19 infection (26, 30, 31).

The mRNA vaccine when first developed was considered safer than inactivated vaccines as it is noninfectious and there is no potential risk of infection (32). However, in our study, the mRNA Pfizer-BioNTech vaccine showed a statistically significant higher percentage of adverse effects compared to the inactivated Sinopharm vaccine but 53.7% reported the adverse effects among the mRNA Pfizer-BioNTech vaccine recipients were local adverse effects. The mRNA Pfizer-BioNTech vaccine recipients also reported a greater number of side effects compared to inactive Sinopharm vaccine recipients. A study that has compared the Sinopharm and Pfizer-BioNTech vaccines has reported more moderate to severe adverse effects after the Pfizer-BioNTech vaccination (33) and another has reported a similar higher percentage of local adverse effects with the Pfizer-BioNTech vaccination than systemic adverse effects (26).

While the percentage of side effects reported did not vary with the number of doses among the inactive Sinopharm vaccine recipients, it was observed that among the mRNA vaccine recipients, the number of adverse effects reported after the second dose was 2.6 times higher than after the first dose of the vaccine. Furthermore, local side effects were reported after the first dose and more systemic side effects were reported after the second dose, which was in agreement with the findings reported in a survey conducted in the United Kingdom (26).

This study showed that among the inactive Sinopharm vaccine recipients, individuals in the younger age group reported significantly higher side effects than the older age groups. Among the mRNA vaccine recipients, individuals in the 35–54-year age group reported more adverse effects than the older individuals but the difference was not statistically significant and the younger individuals (<35 years) reported statistically significantly lesser side effects than the older individuals. On the contrary, another study published on the mRNA vaccine showed that younger age was associated with greater odds of adverse effects. This might be due to the difference in the age group among which the study was conducted as most of the study participants of this study were between 38 and 66 years (34). Now that COVID-19 vaccination for children is recommended, a similar survey is needed to understand the response of the younger age group, especially that in this study we found that among the Sinopharm vaccine recipients, the younger age group of <35 years reported more side effects than among those in the age group of ≥35 years.

In our survey, only 5% of the adverse effects required consultation with a doctor or treatment at the hospital and more than 80% of the participants reported a severity score of <5 supporting the fact that most adverse effects following vaccination were mild in nature and self-limiting.

The strength of this study is the comparison of the adverse effects between inactive and mRNA COVID-19 vaccines, the availability of both inactive Sinopharm and mRNA Pfizer-BioNTech vaccines in the UAE allowed cross-vaccine comparison in the same population. To the best of our knowledge, this is the first study to do a detailed comparison of these two different types of vaccines with a relatively large sample size. Another strength of this study is that the information bias was controlled to a large extent by cross-verification. Among all the participants who were contacted through a telephone interview, the survey details on the side effects and medical advice or treatment of the same were verified using the electronic medical records (EMR). The highly electronically integrated healthcare system in Abu Dhabi ensures easy accessibility for researchers to report more accurate and comprehensive data. Our study also has some limitations as the survey was on voluntary basis individuals who are more concerned about their health or have better health-seeking behaviors are most likely to participate in these surveys creating a participant bias. The results of the study are based on survey-based results, so with any other cross-sectional study, the results do not allow for causality interpretation.

In conclusion, the adverse effects of both the inactivated and mRNA vaccines developed mostly within 24 h of vaccination and about 95% were mild requiring no or home-based treatment. The adverse effects are more likely to be systemic side effects and younger individuals, females, and people with comorbidities are more likely to report adverse effects following inactivated Sinopharm vaccine. Among the mRNA Pfizer-BioNTech vaccine recipients, the adverse effects are more likely to be local after the first dose and systemic after the second dose and observed more among people with associated comorbid conditions and with a previous history of COVID-19 infection.

Thus, most adverse effects reported are mild and this public knowledge on the nature of side effects and the factors associated with greater odds of side effects would instill confidence and overcome vaccine hesitancy among people and enhance vaccine coverage which is the need of the hour.

## Data Availability Statement

The data supporting the conclusion of this article is available with the corresponding author, which shall be provided on approval upon request.

## Ethics Statement

The studies involving human participants were reviewed and approved by Institutional Review Board, Department of Health, Abu Dhabi, UAE. The patients/participants provided their informed consent to participate in this study.

## Author Contributions

SG, LK, and WZ: conception, design of work, and acquisition. MM, NM, MS, AS, HE, RM, NS, KS, and AF: design of work and acquisition. FC, SG, LK, and WZ: analysis. NK, KW, FC, SG, LK, WZ, and FA: interpretation of data. NK, KW, SG, LK, WZ, and FA: drafting and substantively revising the manuscript. All authors read and approved the final manuscript.

## Conflict of Interest

SG, FC, KW, FA, and WZ were employed by G42 Healthcare. SG and WZ was employed by Insights Research Organization and Solutions. MM, NM, MS, AS, HE, RM, NS, KS, and AF were employed by Ambulatory Healthcare Services, SEHA. The remaining authors declare that the research was conducted in the absence of any commercial or financial relationships that could be construed as a potential conflict of interest.

## Publisher's Note

All claims expressed in this article are solely those of the authors and do not necessarily represent those of their affiliated organizations, or those of the publisher, the editors and the reviewers. Any product that may be evaluated in this article, or claim that may be made by its manufacturer, is not guaranteed or endorsed by the publisher.
