# Supplementary material for: Vaccine Side Effects Following COVID-19 Vaccination Among the Residents of the UAE—An Observational Study
Source: Front Public Health. 2022 May 6;10:876336. doi: 10.3389/fpubh.2022.876336 (PMC9120526; doi:10.3389/fpubh.2022.876336)

Table S1: Demographical variables of the study participants

Variables		n	Percentage %
**Age group**	<35 years	599	31.9%
	35-54 years	940	50.1%
	≥ 55 years	339	18.1%
**Gender**	Female	1151	61.3%
	Male	727	38.7%
**Educational status**	Graduate/Post graduate degree	1153	61.4%
	High school / Diploma	429	22.9%
	Less than high school	162	8.6%
	No formal education	134	7.1%
**Income**	≤ 10,000 AED	572	38.6 %
	10,000 and above	969	61.4%
	Did not disclose	519	27.6%
**Nationality**	Emirati	909	48.4%
	Non-Emirati	969	51.6 %
**Comorbidities**	With comorbidities	526	28%
	No comorbidities	1352	72%
**Previous COVID-19 infection**	Yes	332	17.7%
	No	1546	82.3%
**Type of vaccine**	Sinopharm	941	50.1%
	Pfizer	890	47.4%
	Others	47	2.5%
**Adverse effects**	Yes	1217	64.8%
	No	661	35.2%
**Total**		1878	100%

Table S2: Local and systemic side effects

Side effects (n = 1878)		n (%)
**Local**	Pain at the site of the vaccine	882 (47)
	Redness	36 (1.9)
	Itch	31 (1.7)
	Enlarged/swollen lymph nodes	10 (0.5)
**Systemic**	Drowsiness and Fatigue	530 (28.2)
	Muscle and Joint pain	433 (23.1)
	Headache	332 (17.7)
	Fever	270 (14.4)
	Chills	98 (5.2)
	Feeling dizzy	70 (3.7)
	Nausea	61 (3.2)
	Difficulty in sleeping	50 (2.7)
	Decreased appetite	46 (2.4)
	Flu-like symptoms	39 (2.1)
	Muscle weakness	38 (2)
	Heavy sweating	36 (1.9)
	Abdominal pain	34 (1.8)
	Feeling nervous	10 (0.5)
	Rash	7 (0.4)
	Constipation	7 (0.4)
	Gastritis	7 (0.4)
	Vomiting	5 (0.3)
	Change or loss of the ability to taste	4 (0.2)
	Temporary relaxation of one side of the face	1 (0.1)

Table S3: Association of the adverse effects with the type of vaccine

	Sinopharm	Pfizer-BioNTech	Odds ratio	P value
Adverse effects	574 (61.0%) n = 941	610 (68.5 %) n = 890	1.39 (1.14-1.68)	<0.001
Local symptoms	393 (41.8%) n = 941	478 (53.7%) n = 890	1.61 (1.34-1.94)	<0.001
Systemic symptoms	417 (44.3%) n = 941	432 (48.5%) n = 890	1.18 (0.98-1.42)	0.070
Adverse effects after the first dose	214 (37.3%) n = 574	103 (16.9%) n = 610	2.92 (2.23-3.83)	<0.001
Adverse effects after the second dose	208 (36.2%) n = 574	268 (43.9%) n = 610	1.38 (1.09-1.74)	0.007
Adverse effects after both the doses	128 (22.3%) n = 574	232 (38.0%) n = 610	2.14 (1.66-2.76)	<0.001
Local adverse effects after the first dose	142 (66.4%) n = 214	79 (76.7%) n = 103	1.67 (0.97-2.86)	0.062
Local adverse effects after the second dose	133 (63.9%) n = 208	193 (72.0%) n = 268	1.45 (0.98-2.14)	0.061
Systemic effects after the first dose	168 (78.5%) n = 214	66 (64.1%) n = 103	2.04 (1.22-3.43)	0.006
Systemic effects after the second dose	150 (72.1%) n = 208	232 (86.6%) n = 268	2.49 (1.57-3.96)	<0.001
Adverse effects that required consultation/ hospitalization	27 (4.7%) n = 574	32 (5.3%) n = 610	1.12 (0.66-1.90)	0.668

*Adverse effects: For Sinopharm 24 people (4.2%, n=574) didn't specify when they had these symptoms (after first, second or after both doses)

For Pfizer 7 people (1.1%, n=610) didn't specify when they had these symptoms (after first, second or after both doses)

Table S4: Number of side effects reported based on age group and type of vaccine

Age group	Vaccine (n)	Number of side effects reported		Adverse effects (%)	Odds ratio	P value
		1-5	>5			
18-34 years	Sinopharm (366)	195 (53.3)	34 (9.3)	229 (62.6)	1.039 (0.923-1.840)	0.078
	Pfizer-BioNTech (210)	102 (48.6)	16 (7.6)	92 (43.8)		
35-54 years	Sinopharm (505)	287 (56.8)	26 (5.1)	313 (62)	1.441 (1.752 - 1.184)	<0.001
	Pfizer-BioNTech (413)	231 (55.9)	73 (17.7)	304 (73.6)		
≥ 55 years	Inactive Sinopharm (70)	31 (44.3)	1 (1.4)	32 (45.7)	1.835 (1.382-2.436)	<0.001
	Pfizer-BioNTech (267)	164 (61.4)	24 (9)	188 (70.4)		

Table S5: Treatment for side effects post vaccination based on age group

Age group	Vaccine (n)	Treatment for adverse effects		Odds ratio	P value
		No treatment/home based treatment	Doctor consultation or a hospital-based treatment		
18-34 years	Sinopharm (222)	208 (93.7)	14 (6.3)	0.525 (0.169-1.635)	0.525
	Pfizer-BioNTech (117)	113 (96.6)	4 (3.4)		
35-54 years	Sinopharm (300)	289 (96.3)	11 (3.7)	1.490 (0.679-3.268)	0.318
	Pfizer-BioNTech (298)	282 (94.6)	16 (5.4)		
≥ 55 years	Inactive Sinopharm (30)	28 (93.3)	2 (6.7)	0.960 (0.230-4.519)	0.958
	Pfizer-BioNTech (187)	175 (93.6)	12 (6.4)		

Figure S1: Severity of side effects

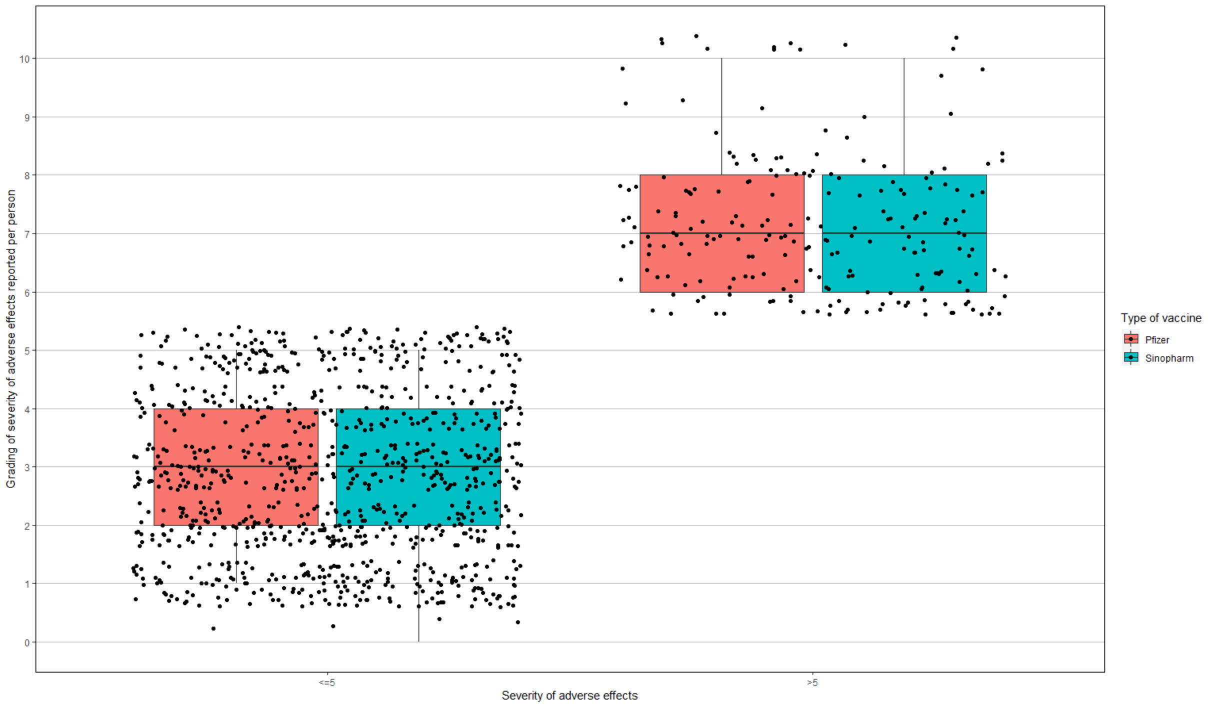

Supplement: Supplementary file 1 [file Table_1.pdf]
